# Development of Nano-SiO_2_ and Bentonite-Based Mortars for Corrosion Protection of Reinforcing Steel

**DOI:** 10.3390/ma12162622

**Published:** 2019-08-17

**Authors:** Venura Kiloshana Karunarathne, Suvash Chandra Paul, Branko Šavija

**Affiliations:** 1 Discipline of Civil Engineering, School of Engineering, Monash University Malaysia, Bandar Sunway 47500, Malaysia; 2 Department of Civil Engineering, International University of Business Agriculture and Technology, Dhaka 1230, Bangladesh; 3 Microlab, Faculty of Civil Engineering and Geosciences, Delft University of Technology, 2628CN Delft, The Netherlands

**Keywords:** corrosion, cracking, nano-SiO_2_, bentonite, mass loss, durability

## Abstract

In this study, the use of nano-silica (nano-SiO_2_) and bentonite as mortar additives for combating reinforcement corrosion is reported. More specifically, these materials were used as additives in ordinary Portland cement (OPC)/fly ash blended mortars in different amounts. The effects of nano-silica and bentonite addition on compressive strength of mortars at different ages was tested. Accelerated corrosion testing was used to assess the corrosion resistance of reinforced mortar specimens containing different amounts of nano-silica and bentonite. It was found that the specimens containing nano-SiO_2_ not only had higher compressive strength, but also showed lower steel mass loss due to corrosion compared to reference specimens. However, this was accompanied by a small reduction in workability (for a constant water to binder ratio). Mortar mixtures with 4% of nano-silica were found to have optimal performance in terms of compressive strength and corrosion resistance. Control specimens (OPC/fly ash mortars without any additives) showed low early age strength and low corrosion resistance compared to specimens containing nano-SiO_2_ and bentonite. In addition, samples from selected mixtures were analyzed using scanning electron microscopy (SEM) and energy-dispersive X-ray spectroscopy (EDX). Finally, the influence of Ca/Si ratio of the calcium silicate hydrate (C-S-H) in different specimens on the compressive strength is discussed. In general, the study showed that the addition of nano-silica (and to a lesser extent bentonite) can result in higher strength and corrosion resistance compared to control specimens. Furthermore, the addition of nano-SiO_2_ can be used to offset the negative effect of fly ash on early age strength development.

## 1. Introduction

Reinforced concrete (RC) is the most widely used construction material in the world. It is cheap, widely available, and durable. In general, steel reinforcement inside the concrete is protected from active corrosion by a passive layer which spontaneously forms on its surface in the alkaline environment [1,2,3]. However, the passive layer may breakdown either due to chloride ingress, when a sufficient amount of chloride ionsreaches the reinforcement [4]; or due to carbonation of the concrete cover [5], when the alkalinity of the concrete pore solution is lost [6]. Once the passivity is lost, active corrosion of the reinforcement starts. Since rust occupies a larger volume compared to the parent steel, its formation will cause stresses in the surrounding concrete, leading to cracking and spalling of the cover [7,8]. The occurrence of damage in the concrete cover will lead to even faster deterioration and corrosion [9]. Corrosion of reinforcement causes more problems than cover cracking alone: it causes a reduction in the effective area of reinforcing steel, thereby lowering the load bearing capacity of the structural member and increasing structural deflections [10]; it affects the bond between steel and concrete [11,12]; and it can reduce the ductility of steel reinforcement, leading to a more brittle behavior [13]. However, in general, signs of deterioration (such as cover cracking or spalling) are visible before serious structural issues arise. In practice, therefore, the initiation of corrosion and occurrence of cover cracking should be delayed in order to increase the service life of RC structures.

At present, various methods are available for delaying the onset of reinforcement corrosion in RC structures. The most common methods are increasing the concrete cover depth and quality, thereby increasing the time needed for chloride ions or CO_2_ to penetrate to the level of reinforcement [1]. In harsh environmental conditions, however, additional protective measures might be needed. These additional measures could either be used in the concrete itself to reduce its permeability or directly applied on the steel reinforcement. Most common measures include the use of sealants and membranes on the reinforcement, admixtures in concrete (e.g., corrosion inhibitors [14]), surface coating (e.g., hydrophobic membranes), and cathodic protection [15]. These corrosion protection techniques can also be split into twocategories: mechanical and electrochemical corrosion protection methods. Mechanical methods prevent direct contact of the rebar with chlorides, oxygen and moisture by acting as a physical barrier. These physical barriers include sealers, membranes, various coatings and overlays applied on the steel rebar [16]. On the other hand, electrochemical methods interfere with the corrosion process by altering the corrosion cell. The most common electrochemical method, cathodic protection, stops the corrosion of the metal surface by making it a cathode of an electrochemical cell [17,18,19]. In practice, however, mechanical methods are (still) more desirable due to ease of application and greater confidence of design engineers. Therefore, developments in mechanical methods are still ongoing. One of the possibilities is the utilization of nanoparticles in concrete to improve its quality.

Over the past few years, using nanoparticles to modify cementitious composites in order to improve their different properties has gained increased attention [20,21]. Nano-SiO_2_ has been the most studied due to its positive effects on fresh [22] and hardened properties of cementitious materials [23,24]. The ultrafine nature of the nano-SiO_2_ particlesand their high surface area increase their ability to fill in spaces existing between particles in the cementitious mixture, and thus improve strength and durability [25]. In this research, therefore, nanosilica is used as additive to improve the corrosion resistance of reinforced cement-based materials.

Apart from durability, sustainability is an important aspect for cement-based materials in practice. As production of Portland cement is the most polluting element in the supply chain, its partial replacement by industrial wastes or byproducts leads to a lower carbon footprint [26,27,28]. In this study, Portland cement has been partly replaced by class F fly ash. In addition, bentonite (Al_2_H_2_Na_2_O_13_Si_14_) was use as partial Portland cement replacement in some of the mixtures to test its effectiveness as part of the corrosion protection measures. Bentonite is usually used in cement-based materials as a filler. However, the use of bentonite as part of corrosion protection in cement-based material has not been previously reported. In general, use of bentonite as partial replacement of Portland cement in concrete leads to lower cost without affecting the compressive strength [29,30]. Bentonite is a natural pozzolan containing both sodium and calcium ions. According to Memon et al. [29], bentonite incorporation in concrete resulted in low early stage compressive strengths and relatively high permeability but during late curing stages (beyond 28 days), these properties were improved significantly. Therefore, bentonite is used in this study as part of corrosion protection measures to be tested.

The focus of this research is therefore to use the different dosages of nano-SiO_2_ and bentonite as cement replacement in cement and fly ash-based mortar. Accelerated chloride induced corrosion test is used since the natural corrosion process in RC structures is slow and it may take long before visible corrosion occurs. The performance of nano-SiO_2_ and bentonite is then compared the control mix. Steel mass loss, maximum pitting depths in the rebar and corrosion induced crack lengths in mortar specimens are also investigated and discussed in the subsequent sections.

## 2. Materials and Methods

### 2.1. Mixture Design

Mixture designs for the control, nano-SiO_2_ and bentonite specimens are shown in Table 1. In all mixtures, 30% by weight of ordinary Portland cement (OPC) was replaced by Class F type fly ash. The particle size and specific surface area of the nano-SiO_2_ and bentonite used in this study were in the ranges of 20–30 nm, 1–5 μm, and 180–600 m^2^/g, 35–60 m^2^/g, respectively. Both nano-SiO_2_ and bentonite were used to replace a part of Portland cement at the dosages of 2%, 4% and 6% of cement weight. It is also worth mentioning that for better distribution, nanoparticles were separately mixed with water and added during the mixing process of all mixes. Mini slump cone test was used to measure the flow behavior of each mixes.

### 2.2. Specimen Preparation

Compressive strength was determined using 50 mm cubic samples. Specimens were demolded 24 h after casting. A minimum of three specimens for each mix type were prepared and water cured (i.e., at 100% relative humidity)until testing at 7, 14, 28 and 56 days.

**Figure 1:**
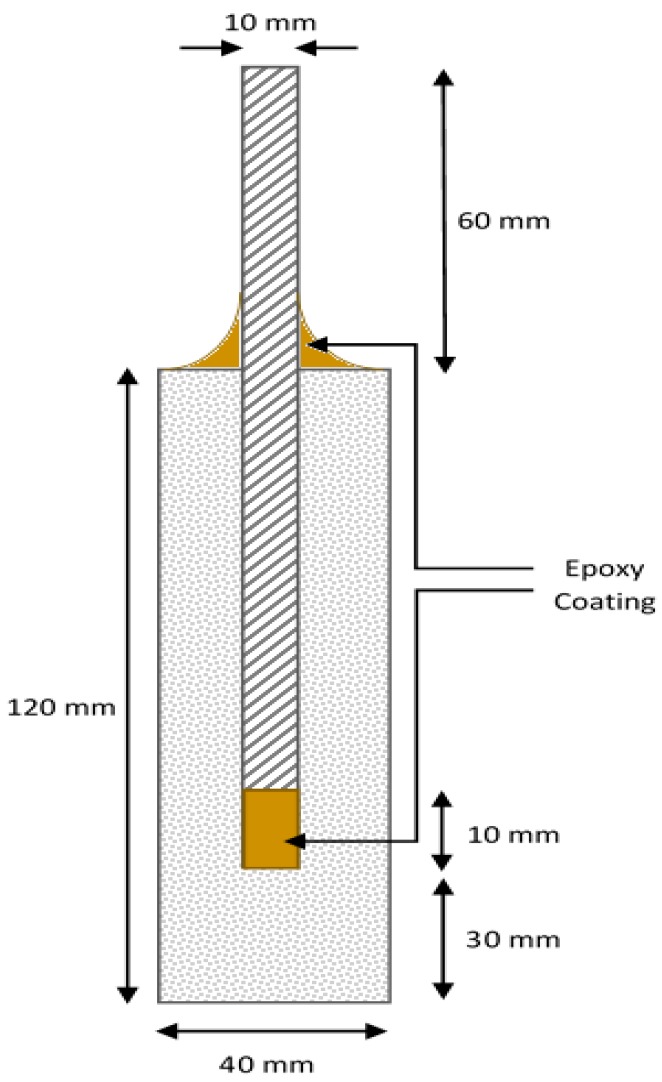
Schematic representation of the reinforced mortar sample used in accelerated corrosion testing.

As mentioned earlier, accelerated corrosion testing was used in this study to examine the performance of nano-SiO_2_ and bentonite mixed mortar specimens compared to control specimens. Cylindrical mortar specimens with 15 mm cover depth, 40mm diameter, and 120mm length were used in the corrosion tests, as illustrated in Figure 1. A 150 mm long mild steel bar with a diameter of 10mm (grade 460 MPa) was used as reinforcement. Prior to embedding in the mortar, surface of each steel rebar was cleaned and its weight was recorded. The intersection between cement surface and rebar was sealed using an epoxy paint applied at height of 10 mm to prevent crevice corrosion. After casting, specimens were demolded after 24 h and cured in water for 7 days before being subjected to accelerated corrosion.

### 2.3. Accelerated Corrosion Testing

**Figure 2:**
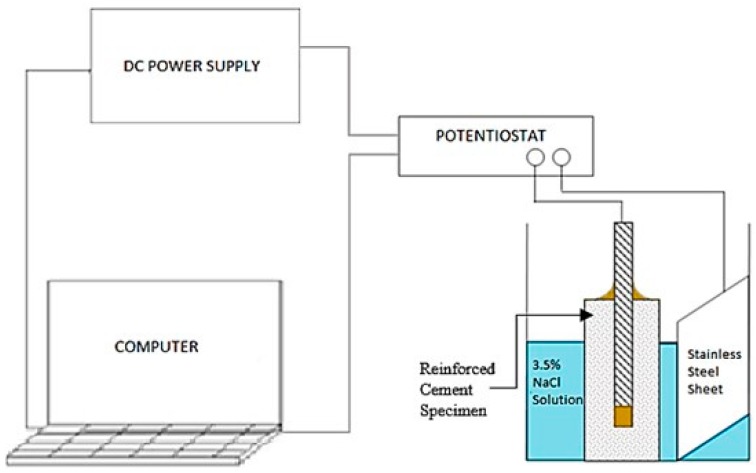
Experimental setup of the potentiostat used in measuring the rate of corrosion (adapted and modified from [35]).

For accelerated corrosion test, an experimental setup was used which consisted of a direct current (DC) power supply, a stainless-steel plate (as the counter electrode) and the steel rebar embedded in the mortar specimen (as the working electrode) to form a potentiostat circuit to obtain the corrosion currents. The stainless steel sheet here was used to induce the polarization potential from the potentiostat to the steel rebar [31]. NaCl (3.5%) solution was also used to create a corrosive environment, as shown in Figure 2. A constant voltage of 15 V was supplied in the circuit to set an accelerated corrosion. A schematic representation of the accelerated corrosion is shown in Figure 2. Although, in reality, corrosion of steel reinforcement is a slow process compared to accelerated testing conditions adopted herein, this test set up allowedcomparing the performance of specimens with different compositions within an acceptable timeframe. This test setup has therefore been commonly used in the literature [32,33,34].

The experiment was carried out for 3 days (72 h) and corrosion current measurements were recorded every 3 seconds. By applying the Faraday’s law, the corrosion current measured by the potentiostatwasthen converted into corrosion mass loss. Equation (1) below represents the Faraday’s Law used for the calculation of the mass loss where *∆m*=mass loss of steel (g); *M*= atomic mass of Fe (56 g/moL); *I*= corrosion current (A); *∆t*= time interval (s); *Z*= valency of Fe (2); and *F*= Faraday’s constant (96,500 A/s) [32]:

(1) $$\Delta m=\frac{MI\Delta t}{ZF}.$$

In this work, mass loss calculated using Faraday’s law wascompared to the measured mass loss of each individual rebar at the end of the test. To achieve this, after the corrosion test was finished, all specimens were broken manually using a hammer. The corroded rebars were collected and cleaned with a wire mesh brush to remove the corrosion products from the surface, and weighed. The actual mass loss was then determined based on the difference between the weight of the cleaned rebar after the corrosion test and the initial rebar weight. A minimum of two specimens per mixture were tested, and the average mass loss is reported herein.Note that rust was removed manually, i.e., no acid was used in cleaning of the corroded rebars, which could somewhat underestimate the mass loss.

Finally, to evaluate the microstructure of the mortar specimens, a scanning electron microscope (SEM) was used to obtain images under high magnification. Elemental identification in the mixes was also performed using energy-dispersive X-ray spectroscopy (EDX) analysis. The samples for SEM and EDX were taken from cylindrical specimens used in the corrosion test. After 72 h of accelerated corrosion testing, these samples were collected by cutting from cylindrical specimens of control, 4% nano-SiO_2_and 4% bentonite mixes only. All samples were first coated with a layer of platinum by applying sputter current of 30 mA and sputter time of 40 s via Quorum Q150R S machine (Quorum Technologies Ltd, East Sussex, UK) and the SEM test was conducted using field-emission scanning electron microscope (FE-SEM, Hitachi, Tokyo, Japan). EDX test was conducted using X-max Horiba to perform the elemental analysis of the specimens.

## 3. Results and Discussion

### 3.1. Slump Flow

**Figure 3:**
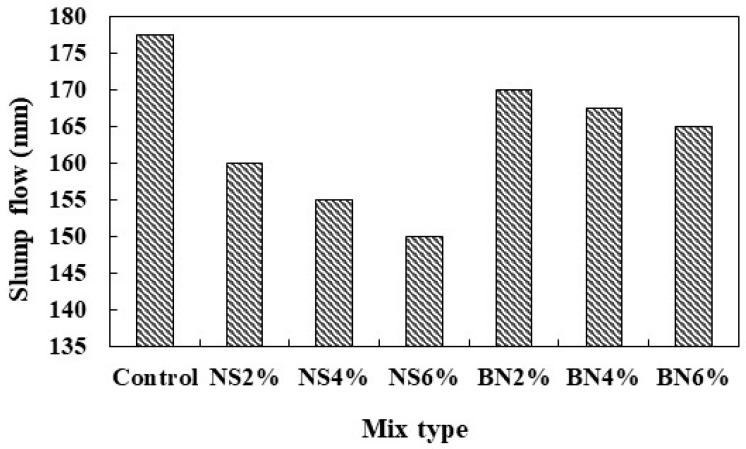
Slump flow measurements for mortar mixtures with different amounts of nano-silica and bentonite.

The workability of all fresh mortar mixes was checked tested using a mini slump cone according to ASTM C230. The results are given in Figure 3. It can be seen that the maximum slump flow was found in the control mix with an average value of 177.5 mm while the minimum slump flow was observed in the mix containing 6% nano-SiO_2_ with an average slump flow of 150 mm. The slump flow values of nano-SiO_2_ mixes in general were lower compared to both the control and the bentonite mixes. The reason for the significant drop of slump flow values for the samples containing nano-SiO_2_ particles is probably their high surface area as compared to bentonite and cement, resulting in increased water demand [36]. Typically, higher surface area initiated to higher amount of absorbed water from the mix and reducing the amount of free water hence reducing the workability of the samples [25].

### 3.2. Compressive Strength

**Figure 4:**
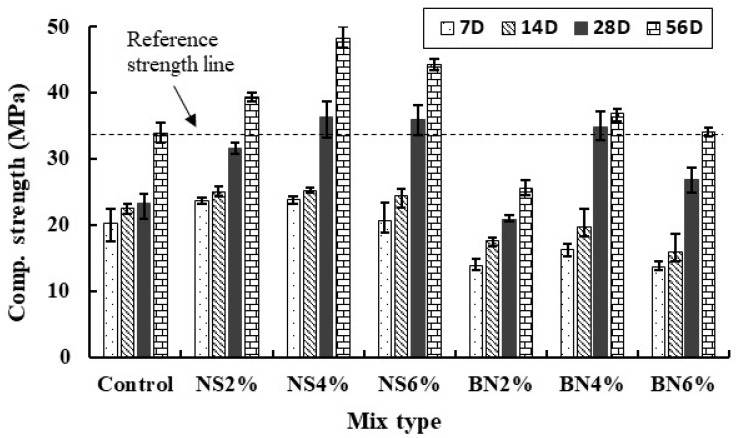
Strength development for mortar mixtures with various amounts of nano-silica and bentonite.

Compressive strength development of all mixes at 7, 14, 28 and 56 days is presented in Figure 4. For comparison, reference strength (considered for 56 days) line is drawn with respect to the control sample is indicated by a dotted line in Figure 4. Average compressive strength was calculated from a minimum of three specimens for each mixture. Results reported in Figure 4 show that the inclusion of nano-SiO_2_ in the cement mortar enhances the compressive strength compared to both control and bentonite mixes. This can be attributed to the pozzolanic reaction caused by nano-SiO_2_. Nanoparticles can physically and chemically affect the properties of cementitious composites. Physical effects involve filling of the micro pores present in the microstructure resulting in higher compressive strength [37]. Chemical effects include the promotion of pozzolanic reaction on their surfaces by the nanoparticles: this leads to formation of calcium-silicate-hydrate (C-S-H) gel. This C-S-H gel will grow on the surfaces of the nanoparticles and then serve as the nucleation sites and produce a denser and stronger matrix. Nanoparticles also have the ability to reduce the quantity and grain size of the calcium hydroxide (Ca(OH)_2_) by reacting with (Ca(OH)_2_) to form C-S-H gel [24] and enhance the crystal orientation of Ca(OH)_2_ between the hardened cement-fly ash paste and the aggregate [38].

Since the particle size of nano-SiO_2_ is finer compared to that of the cement, fly ash and bentonite, therefore, nano-SiO_2_ particles would have filled more smaller pores present in the matrix. This results in higher strength compared to both control and bentonite mixes. Compared with the control mix, inclusion of 4% nano-SiO_2_ and bentonite in the mixes resulted in about 57% and 50% higher strength at 28 days. At 56 days, these values were about 42% and 9%, respectively. The results also indicate that the mix design containing 4% of nano-SiO_2_has the highest compressive strength compared to 2% and 6% dosages. This can be attributed to the fact that below the certain dosages limit, nanoparticles may enable less nucleation sites therefore resulting in lower strength. On the other hand, higher dosages of nanoparticles containing high surface energy can lead to agglomeration and uneven dispersion of the nanoparticles in the matrix, leading to the formation of weak zones in the mortars. Higher dosages of nanoparticles increase the specific surface area which may absorb more water from the mixture and thus the specimen may suffer excessive self-desiccation and cracking [39,40]. This phenomenon can be valid both for nano-SiO_2_ and bentonite specimens. Therefore, a similar strength development pattern is found in these mortar mixes.

Finally, an increase in the compressive strength in all samples was observed with prolonged curing 28 days to 56 days. It is also interesting to notice that the control mixes with 30% of cement replaced by the fly ash show slower strength development from 14 days to 28 days while in all nano-SiO_2_ and bentonite mixes this trend was opposite. This implies that the addition of nanoparticles such as nano-SiO_2_ and bentonite can accelerate the hydration process of cement and fly ash-based mortar and provide early high strength. This was also reported in the previous studies with nanoparticles [21]. This can be attributed to the accelerating effect of nano-silica on cement hydration [36]. Therefore, in the cementitious materials with high volume of fly ash, optimum dosages of nano-SiO_2_ and bentonite can be considered if early strength is required. It has been reported, however, that the addition of nano-silica in fly ash containing mixtures can hinder the pozzolanic reaction of the binder at later ages due to early consumption of Ca(OH)_2_ [41]. This effect was not observed in the current study, since it seems to be of importance at even later ages (compared to 56 days in this study). Nevertheless, this effect needs to be considered in practical applications.

### 3.3. Mass Loss Due to Corrosion

**Figure 5:**
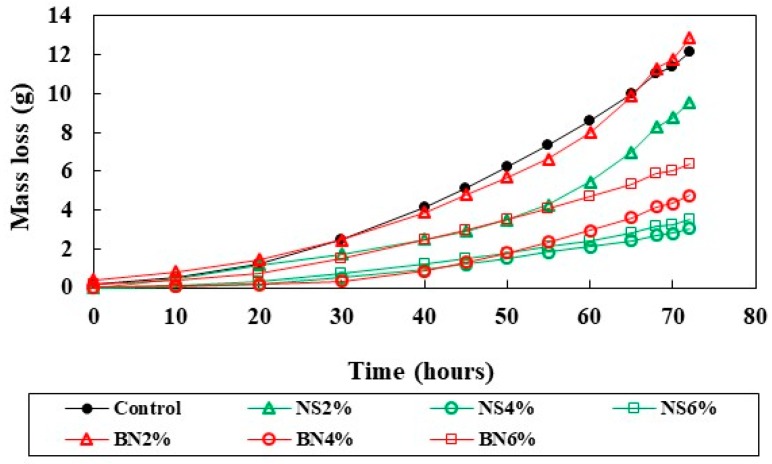
Loss calculated using Equation (1) for different mixtures.

**Figure 6:**
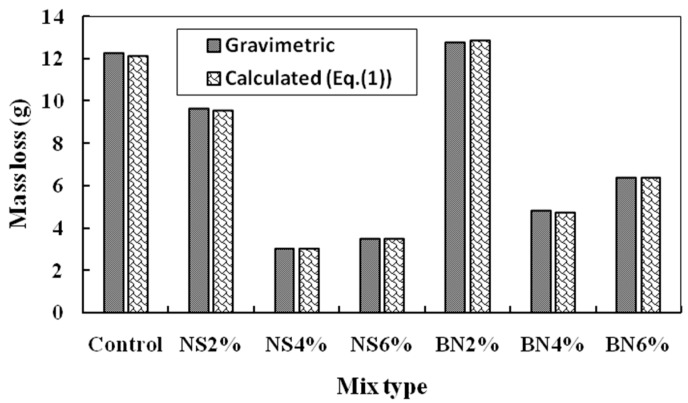
Comparison of between the actual mass loss (gravimetric) and the mass loss calculated using Faraday’s law (Equation (1)) for all mix designs after 72 h.

Corrosion current values obtained after 72 h of corrosion testing using the potentiostat were analyzed and the resulting mass loss values of the rebar were calculated using the Faraday’s Law (Equation (1)). These average mass loss values obtained from a minimum of two specimens for each mix design after 72 h of accelerated corrosion test is shown in Figure 5. The comparison between the experimental and actual mass loss of the steel rebars is also shown in Figure 6.

The specimens containing 4% nano-SiO_2_ displayed the lowest mass loss whereas the specimens containing 2% bentonite and the control samples showed the highest values. Most nano-SiO_2_ containing specimens in general showed lower mass loss compared to that of the bentonite and control samples. As mentioned earlier, due to the high surface area of the nano-SiO_2_ particles and its ability to fill nano-sized pores within C-S-H, the nano structure of the mixtures containing nano-SiO_2_is strengthened which results in enhanced properties including lower permeability and higher electrical resistivity of the specimens [42]. This lower permeability and high electrical resistivity of nano-SiO_2_ specimens may lower the rate of ion transfer in the electrochemical cell thus lowering the corrosion rate. In an earlier study [37] it was also found that the cement mortar specimens containing nano-SiO_2_ have higher electrical resistance, i.e., less ion movement than the control specimen. Addition of nano-SiO_2_ in the mix may consume most of the Ca(OH)_2_ leaving a lower amount of calcium to silicate (Ca/Si) ratio of the C-S-H which is an important parameter for strength development in the cementitious materials [21]. Bentonite specimens with 4–6% also showed lower mass loss compared to control samples but higher compared to all specimens with nano-SiO_2._ Bentonite particles are relatively larger compared to the nano-SiO_2_ and this may contribute to higher permeability and lower electrical resistivity as compared to nano-SiO_2_ mixed specimens.

Figure 6 gives a comparison of the mass loss calculated using Faraday’s law (Equation (1)) and the actual mass loss measured using the gravimetric method. A low difference between the gravimetric and the calculated mass loss of steel rebars is also observed. as shown in Figure 6. This shows that the accelerated corrosion testing as used in this work is reliable.

**Figure 7:**
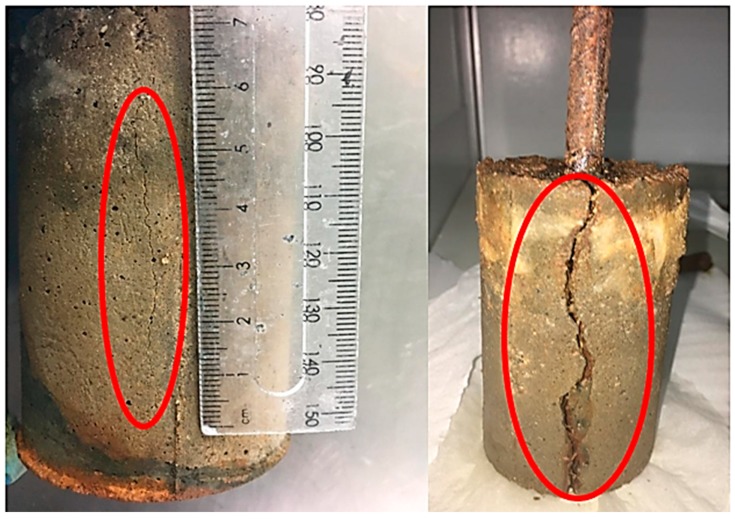
Visible cracks in 4% nano-SiO_2_ specimen (**left**) and control specimen (**right**).

Corrosion induced cracks in the specimens were also investigated. Table 2 shows the average crack length in the specimens after 72 h of testing. In most cases, a single crack developed on one side of the specimens as shown in Figure 7. The crack length was then measured with a ruler and the average crack length from the two specimens for each mortar mixwas calculated and reported in Table 2. It is also noted that, in most specimens, cracks were visible already after 24–48 h of accelerated corrosion testing. Note that crack widths were not measured in this research work. The shortest was found in the specimens containing 4% of nano-SiO_2_. Both the control and the 2% bentonite specimens showed longer cracks compared to other specimens. In these series, cracks were visible along the entire length of the specimen as shown in Figure 7. A comparison of crack development in nano-SiO_2_ (4% dosage) and control specimens can be seen in Figure 7. Nevertheless, this observation further justifies the fact that nano-SiO_2_ in general shows better corrosion protection properties compared to both bentonite and control sample.

**Figure 8:**
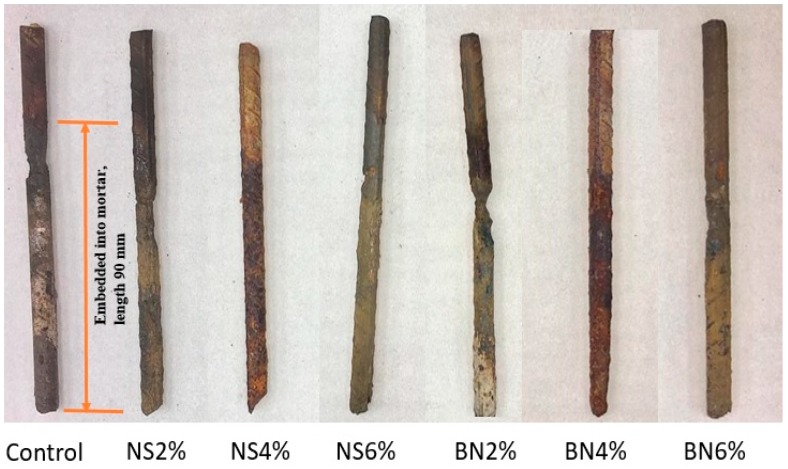
Rebars of all mixtures after cleaning.

Figure 8 represents the visual observation of the corroded steel rebar of each mix design. As already discussed, after corrosion testing all specimens were broken and steel rebars collected to measure the actual (gravimetric) mass loss and compare with the mass loss calculated using Equation (1). Steel rebars were cleaned using wire mesh brush so that no substantial amount of mortar sticks with them. Also, no chemical was used to clean the steel rebars as it was reported that the variation between the experimental and actual mass loss can significantly be different when chemical is used to clean the steel rebar [43]. Thisis due to fact that some chemicals not only remove the rust but also result in partial dissolution ofvirgin steel. In most cases, general corrosion over the crack lengths was observed in the rebars. However, concentrated corrosion was also visible in few specimens at different points of the rebar. This could be due to the existence of wider cracks and thus allowing the transport of NaCl solution on the surface of the rebars [44]. Thepitting depth in the rebars was also measured using a digital caliperwith an accuracy of 0.1 mm. In this regard, pitting depths in each rebars surface weremeasured at several points and their average depths are reported in Table 3. Similar to mass loss, very low pitting was also observed in specimens with 4% nano-SiO_2_. Therefore, from the results obtained in this research it can deduced that if the optimum dosage of nano-SiO_2_ is used in the mortar mix, it can significantly extend the service life of RC structures in areas with chloride exposure.

### 3.4. Microstructural Analysis

**Figure 9:**
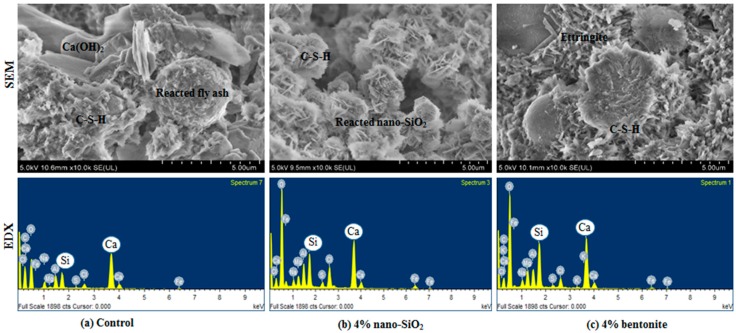
Scanning electron microscopy (SEM) images and energy-dispersive X-ray spectroscopy (EDX) element analysis of different mortar mixes.

To understand the microstructure, samples for scanning electron microscopy were collected from the control, 4% nano-SiO_2_ and 4% bentonite specimens. The selection of the specimens for SEM test was based on the performance of the mixes in compressive strength test. As already discussed, 4% nano-SiO_2_ and 4% bentonite mixes showedthe best performance among nano-SiO_2_ and bentonite mixes, respectively. From the SEM images shown in Figure 9, reacted fly ash particles, presence of ettringite, Ca(OH)_2_ molecules and C-H-S molecules were observed in different mortar mixes. Generally, nanoparticles can enhance the microstructure of the hardened mortar as they can promote the generation of C-S-H gel via accelerated hydration process [45,46]. In the nano-SiO_2_ sample (Figure 9b), C-S-H gel in colloidalform was observed, whereas some needle-like ettringite and hexagonal flakes of large Ca(OH)_2_ crystals were observed in both control and bentonite samples in Figure 9a,c.

Furthermore, the calcium to silicon (Ca/Si) ratio for each sample was measured in each specimen using EDX spot analysis. The Ca/Si ratio measurements for the control, 4% nano-SiO_2_ and 4% bentonite samples were 0.89,0.71 and 0.80, respectively. This indicates that when the Ca/Si ratio is reduced, the compressive strength of that particular sample increases. This is in accordance with the literature [47]. It also worth mentioning that the Ca/Si ratio of the C-S-H can range from 0.67 to 2.0 and thus, from the chemical point of view, it is one of most crucial factors for strength development in the cementitious materials [48].

## 4. Conclusions

The main goal of this research was to compare the performance of mortar mixtures with nano-silica and bentonite to a reference cement-fly ash mixed mortar in terms of compressive strength and corrosion resistance. Experimental results showed that nano-silica and bentonite both can, to different extents, improve corrosion resistance of cementitious mortars. Based on the presented results, the following conclusions can be drawn:

- Addition of nano-SiO_2_ and bentonite increases the compressive strength of the mortar. However, strength may decrease if the dosages higher than a certain amount are used. In this study, at 56 days, 4% nano-SiO_2_ and bentonite dosages (% replacement by cement weight) showed about 42% and 8.5% higher compressive strengths, respectively, than the control mortar mix;
- Mortar mixes with 30% of cement replacement by the fly ash show higher strength development at longer curing period, i.e., at 56 days. This is expected due to slow pozzolanic reaction of fly ash. However, addition of nano-SiO_2_ and bentonite can accelerate the hydration process of cement and fly ash based mortar and provides high early strength;
- Nano-silica and bentonite mixtures had lower Ca/Si ratios (about 25% and 11% lower), corresponding to higher compressive strength compared to control specimens. This is in accordance with known literature;
- Similar to compressive strength, lower steel mass loss due to corrosion was also found in the mortar specimens containing 4% of nano-SiO_2_(about 75% and 60% lower). Similarly, the corrosion induced crack length, width and pitting depth were also lower in 4% nano-SiO_2_ specimens. No improvement in corrosion resistance was found with 2% bentonite dosages. However, with higher dosages, a noticeable difference was found when compared with the control mortar mix.

Further research should be conducted to ascertain the impact of different types of nano-SiO_2_ and bentonite on corrosion of steel reinforcement in cementitious materials. Previous studies have shown that properties of cementitious materials can significantly vary depending on the type of nanoparticles used. In addition, natural corrosion (i.e., not accelerated) testing needs to be performed in order to confirm that the findings from the current study are applicable to real engineering practice.

## Figures and Tables

**Table 1:** Mix designs for control, different dosages of nano-SiO_2_ and bentonite samples.

Materials (kg/m^3^)	Control	NS2%	NS4%	NS6%	BN2%	BN4%	BN6%
Fly ash (class F)	120	120	120	120	120	120	120
Cement (OPC CEM I 42.5)	280	272	264	256	272	264	256
Nano-silica	0	8	16	24	0	0	0
Bentonite	0	0	0	0	8	16	24
Sand	1000	1000	1000	1000	1000	1000	1000
Water	194	194	194	194	194	194	194

**Table 2:** Average crack length of all mixtures after 72 h.

Specimen	Crack Length (mm)						
	Control	NS2%	NS4%	NS6%	BN2%	BN4%	BN6%
1	120	75	60	68.5	100	72.5	90
2	112	70	60	70	105	70	85
Average	116	72.5	60	69.25	102.5	71.25	87.5

**Table 3:** Maximum pitting depths of each mix design.

Mixture	Average Maximum Pitting Depth (mm)
Control	4
NS2%	4
NS4%	1
NS6%	3
BN2%	7
BN4%	4
BN6%	3
